# Draft Genome Sequences of Four Metallo-Beta-Lactamase-Producing Multidrug-Resistant Klebsiella pneumoniae Clinical Isolates, Including Two Colistin-Resistant Strains, from Cairo, Egypt

**DOI:** 10.1128/MRA.01418-18

**Published:** 2019-02-14

**Authors:** Heba Attia, Richard Szubin, Aymen S. Yassin, Jonathan M. Monk, Ramy K. Aziz

**Affiliations:** a Department of Microbiology and Immunology, Faculty of Pharmacy, Cairo University, Cairo, Egypt; b Systems Biology Research Group, Department of Bioengineering, University of California, San Diego, La Jolla, California, USA; c The Center for Genome and Microbiome Research, Cairo University, Cairo, Egypt; Indiana University, Bloomington

## Abstract

The emergence and spread of metallo-beta-lactamase-producing multidrug-resistant Klebsiella pneumoniae are a serious public health threat. Here, we report the draft genome sequences of four K. pneumoniae strains isolated from Cairo, Egypt, including two panresistant colistin-resistant strains. Genome annotation indicated a number of virulence and resistance genes agreeing with observed phenotypes.

## Abstract (precis)

The emergence and spread of metallo-beta-lactamase-producing multidrug-resistant Klebsiella pneumoniae are a serious public health threat. Here, we report the draft genome sequences of four K. pneumoniae strains isolated from Cairo, Egypt, including two panresistant colistin-resistant strains.

## ANNOUNCEMENT

The emergence of multidrug-resistant pathogens, or “superbugs,” poses a potential public health threat as they seriously challenge the treatment of clinical infections. Superbugs cause infections resistant to all beta-lactams and most other antibiotics except colistin and sometimes tigecycline (1). Among multidrug-resistant (MDR) bacteria, the Enterococcus faecium, Staphylococcus aureus, Klebsiella pneumoniae, Acinetobacter baumannii, Pseudomonas aeruginosa, and Enterobacter species (ESKAPE) pathogens (2) are considered the most threatening.

K. pneumoniae, a facultative anaerobic Gram-negative bacterium, causes a wide range of clinical diseases (3) that may be life threatening (4). Resistant strains have swiftly spread worldwide since an initial report in 2008 (5–8), and when colistin resistance was detected in K. pneumoniae (9, 10), they were considered a warning sign of an imminent doomsday scenario.

Here, we report the draft genome sequences of two panresistant Klebsiella pneumoniae strains (SF and SK), which are also colistin resistant, in addition to two other genomes from one panresistant non-colistin-resistant strain (HM) and one strain (SP) that is MDR but sensitive to 10 tested antibiotics (Table 1).

**TABLE 1:** Information related to the sequenced isolates, sequencing, assembly, and annotation results, including predicted antibiotic resistance genes, transporters, and virulence factors^*a*^

Attribute	Analysis or prediction tool	Data for strain:			
		SF	SK	HM	SP
Resistance phenotype^*a*^	Kirby Bauer disk diffusion (24 antibiotics tested)	Panresistant (24/24)^*b*^	Panresistant (24/24)^*b*^	Multiresistant (23/24)^*b*^	Multiresistant (14/24)^*b*^
Colistin resistance	MIC determination	R^*c*^	R	S^*c*^	S
No. of sequence reads	Illumina HiSeq 4000	2.76 × 10^6^	2.38 × 10^6^	2.33 × 10^6^	2.18 × 10^6^
No. of assembled contigs (>500 bp)	SPAdes version 3.10.0 (in PATRIC)^*d*^	352	138	137	93
Genome size (bp)	PATRIC	5,710,047	5,762,786	5,759,337	5,343,887
G+C content (%)	PATRIC^*e*^	56.9	56.85	56.86	57.44
Protein-coding genes	PATRIC	5,932	5,862	5,853	5,248
No. of tRNA genes	PATRIC	65	67	67	68
No. of rRNA genes	PATRIC	6	8	8	7
No. of antibiotic resistance factors	CARD	79	76	76	68
	NDARO	17	19	19	12
	PATRIC	42	39	39	33
No. of transporters	TCDB	593	572	572	567
No. of virulence factors	Victors	155	150	149	145
	PATRIC-VF	116	114	114	112
	VFDB	36	33	33	20

a These predictions were made in the PATRIC portal (15), with reference to various databases, namely, CARD (18), NDARO (19), TCDB (20), victors (21), and VFDB (22).

b Number of antibiotics to which the isolate is resistant (out of total tested).

c R, resistant; S, susceptible.

d SPAdes was used with default parameter on the following two steps: (i) error correction mode only and (ii) assembly mode with mismatch correction.

e All PATRIC analyses were performed by RASTtk on PATRIC database release of July 2018.

The susceptibility of all four strains, isolated from patients in Cairo, Egypt, between 2012 and 2015, was determined by the Kirby-Bauer disk diffusion method (11), and their MICs were determined by the agar dilution method.

The isolated strains were obtained from local hospital laboratories, in which they were biobanked, and regrown on MacConkey agar (for purity checking). Representative colonies were subcultured on LB broth overnight (at 37°C) to be used for DNA extraction. The Wizard SV genomic DNA purification system kit (Promega, Madison, WI, USA) was used to extract genomic DNA from each isolate, and a miniaturized version of the Kapa HyperPlus Illumina-compatible library prep kit (Kapa Biosystems, USA) was used for library generation.

A mosquito high-throughput sequencing (HTS) liquid-handling robot (TTP Labtech, Inc., Melbourn, United Kingdom) was used for 1/10 scale enzymatic fragmentation, end-repair, and adapter-ligation reactions. Sequencing adapters based on the iTru protocol (12) were added in subsequent PCR steps. Amplified and barcoded libraries were then quantified by the PicoGreen assay and pooled in approximately equimolar ratios before being sequenced on an Illumina HiSeq 4000 instrument to >30× coverage.

The Illumina reads were provided as quality-trimmed sequences, whose quality was rechecked and verified by FastQC (13). Reads for K. pneumoniae isolates were submitted to the genome analysis services at PATRIC (14, 15), in which they were assembled into contigs by SPAdes (16) version 3.10.0 and subsequently annotated. The combined size and G+C of assembled contigs (Table 1) were comparable to the other *Klebsiella* genomes in public databases.

PATRIC annotation used the default Rapid Annotations using Subsystem Technology toolkit (RASTtk; July 2018 release) algorithm (17). Many of the annotated genes were homologous to known virulence factors, drug targets, and antibiotic resistance genes (Table 1). Of note, the number of resistance genes was slightly lower in the strain with resistance to the fewest antibiotics (SP) (Table 1).

The presented genome sequences of clinical multidrug-resistant K. pneumoniae strains are among the few sequenced isolates from Egypt to date and should help global efforts to trace and contain the spread of multidrug-resistant pathogens.

### Data availability.

All reads were deposited in the NCBI SRA under BioProject identifier (ID) 493667 (BioSample numbers SAMN10141025 through SAMN10141028). The assembled contigs and their primary annotations were deposited in NCBI as well, under GenBank accession numbers RXLV00000000, RXLX00000000, RXLW00000000, and RXLY00000000 for SF, SK, HM, and SP, respectively.
